# Resistance and the regimen: The microthanatopolitics of Venezuelan antiretroviral scarcity and HIV drug adherence failures

**DOI:** 10.1016/j.socscimed.2024.117177

**Published:** 2024-08-03

**Authors:** Rebecca Irons

**Affiliations:** Institute for Global Health, University College London, London, United Kingdom

## Abstract

The Venezuelan State does not provide adequate antiretroviral therapy (ART) for the population living with HIV, resulting in pharmaceutical scarcity, involuntary treatment pauses, and adherence failures. Such a situation may result in the development of resistance to certain ART drugs, meaning that Venezuelans with HIV may have their treatment options reduced for the remainder of their lives. It can take a number of years for a person to acquire late-stage HIV/AIDS and for death to occur, and so I focus on the microbiological death of CD4 cells over time – a concept I call ‘microthanatopolitics’.

In this paper I argue that the microthanatopolitics of ART scarcity deprives those living with HIV of future treatment options, encourages resistance to ART drugs, and ultimately may contribute towards ill health long after treatment availability changes in Venezuela. To explore this in depth, the paper draws upon 6 interviews with Venezuelan HIV activists in Venezuela (2024), supported by 40 testimonies from Venezuelan migrants living with HIV in Colombia (2021–2024), with and without known ART resistance.

It will be concluded that not only is this an issue for those currently living in Venezuela, but also for migrants and the global HIV response who will suffer from the promotion and circulation of ART-resistant viral strains in the long run. This microthanatopolitics is influenced by both the current Venezuela political system as well as humanitarian aid from the Global North; an important consideration of coloniality in post-colonial Latin America.

## 1 Introduction

It had been five months since Germán had taken any antiretroviral therapy (ART), and what had started as a jagged cough had quickly progressed to a difficult respiratory burden that caused his chest to ache with no respite. His partner Iván was also living with HIV, though had received his diagnosis some years after Germán was first infected and had more favourable CD4 cellular counts when he last visited the hospital. He too was affected by a lack of ART but was showing no troubling signs of late-stage HIV/AIDS yet, unlike Germán. In 2014, ART was still available in the Venezuelan public health system-a situation that would soon change in 2016- however, this did not mean that everyone had access to it. Whilst the pharmaceuticals were technically free of charge, at this point in time Venezuelans needed to pay for routine bloodwork to be undertaken every three months in order to be approved to continue ART treatment. Germán and Iván, both working in a supermarket, could not always afford the trimonthly tests, and so slowly began experiencing gaps in their ART adherence. When Germán eventually became too ill to work the couple felt desperate and fled to Bogotá in neighbouring Colombia, where a local hospital count of CD4 cells below 100mm/3 told his condition had progressed to late stage 3 HIV. Germán was diagnosed with late-stage HIV/AIDS. Although healthcare is not routinely offered to all Venezuelans, the pair were lucky and in Bogotá, Germán’s AIDS-related pneumonia was treated successfully. Both he and Iván were given a month’s supply of ART before being discharged and finding that the Colombian public health system could no longer offer medication due to their residency status. Recounting the story to me in December 2023, Iván could not remember the particular drug combination the hospital had provided, but he was sure that the couple both experienced another few months without ART before eventually finding support from an NGO in the Caribbean city of Santa Marta. Germán’s condition slowly improved, however he found that the drug regimen routinely offered by the NGO was not supporting the increase of CD4 cells as it should. They quickly changed him to another pharmaceutical cocktail, a solution that would not have been available in Venezuela, where instead Germán would have faced a future of ART treatment failure. What can be said of a situation whereby a person living with HIV is unable to access suitable ART and may develop resistance? When I asked these questions to Venezuelan HIV activist Jhonny, his opinion was unequivocable – “the [Venezuelan] government is committing genocide against people with HIV”. In this paper, I will explore this assertion and contextualise ART scarcity within the wider post-colonial, geopolitical context.

Venezuela has recently garnered significant attention as Latin America’s largest ever refugee crisis on record, with 7.7 million people having migrated out of the country since 2015 (UNHCR, 2024). This situation is largely down to the social, political, and economic crisis brought about by national mismanagement and international trade restrictions (Wilson, 2015). The public health system has suffered significantly, at all levels and across all services, with HIV related services and treatments being significantly reduced and at times, absent (Page et al., 2019; Rendon and Schneider, 2018; Pierce, 2017). The government stopped publishing health statistics in 2016 (Johns Hopkins, 2023), however the migrant population with HIV was estimated to be around 8000 in 2019 (UNAIDS, 2021), and an estimated 79,467 Venezuelans are living with HIV in Venezuela as of 2019 (Schreiber, 2019). PAHO has reported a sharp spike in HIV diagnoses as ART has become increasingly unavailable, noting a 24% increase from 2010 to 2016 (when data was last available) (2018). There are a lack of testing kits, meaning that many Venezuelans may be undiagnosed and unaware of their condition (Schreiber, 2019). Further, there is a lack of contraception available (Irons, 2022; Albaladejo, 2018) and so people cannot protect themselves from HIV and other STIs. To illustrate, at its worst, a 36-pack of condoms reportedly cost $USD755 in 2015 Venezuela (Regan, 2015). This occurred in a country where the minimum wage for a public sector worker can be as low as $USD6 per month (Reuters, 2023). The proliferation of HIV without contraception, testing, or ART, not only contributes towards greater disease burden amongst the domestic population and those who migrate, but also adds towards the development of resistant viral strains and a potential dire future of exacerbated treatment options for Venezuelans, even if and when the economy improves. The World Health Organisation have highlighted ART resistance as a global issue of concern, for which international stakeholders need to be united in combating (2022). This Venezuelan legacy could have significant ramifications beyond the country’s borders, and need be seriously addressed.

### 1.1 Microthanatopolitics

In this paper, I will argue that through the handling of the national HIV programme and the approach towards ART, the Venezuelan government (and by extension, the Global North actors implicit in both Venezuela’s crisis and current ART rollout, such as The Global Fund) are not enacting biopolitics, the control over the living, but thanatopolitics, the control over death; a term arising from the Greek *thanatos*, meaning death.

Thanatopolitics was first mentioned by Foucault (1988) in relation to biopolitics, and the term was later developed by the Italian philosopher Agamben (1995), with particular reference to the suffering of the Holocaust. The term ‘thanatopolitics’ is sometimes thought to be synonymous with Mbembe’s (2003) ‘Necropolitics’, from the Greek *nekros*, meaning corpse. Though there are certainly similarities, there is a crucial difference between the ideas that I need to maintain in this paper in order to elucidate the main point, following Troyer’s (2020) explanation:

“Thanatos is death as a concept […] Nekros is a corpse and suggests the dead body in all forms. It is correct to say that both necro and thanato mean “death”, but each word’s use connotes quite specific conditions” (2020: 125).

For necropower to be observed, there arguably must be dead bodies present; if not, and if death is only a potential outcome after a period of time (such as in late-stage HIV/AIDS-related death), it is more appropriate to view this as thanatopolitics, as death looms as a possibility but is not immediately quantifiable. To build on this, unlike Agamben, Mbembe considers racial discrimination and the effects of colonialism to be key factors in the necropower of contemporary states, making necropolitics a very useful framework when considering control of death in the post-colonial Global South, including but not limited to Venezuela. Latin American scholars have linked necropolitics to Quijano’s coloniality of power (Andrade, 2022), whereby colonial hierarchies continue to influence the groups over whom a politics of death is exerted. Andrade notes that necropolitics allows us to frame a politics of death in the Global South (2022: 15). In line with understandings of colonialist influence, this power extends to indigenous peoples (Ruette-Orihuela et al., 2023; Russo Lopes and Bastos Lima, 2020; Smith, 2013), those racialized as non-White (Smith, 2016) and, of particular relevance to this paper, displaced individuals involved in contemporary migrant crises (Estévez, 2021; Trevino-Rangel, 2021; Arias Jr, 2017), and members of the LGBTQ + community (Ribeiro et al., 2021; Caravaca-Morera and Padilha, 2018).

The Venezuelan State has specifically been accused of exerting necropower over its citizens, for example through the extra-judicial execution of marginalised communities, “killable” populations based on race, class and geographic location” (Hanson and Zubillaga, 2021: 190), by the miliary and police. Hanson and Zubillaga argue that necropolitical governance has been dominant during Maduro’s presidency, with necropower accusations extending beyond the cities to the violent exterminations of the Indigenous Yanomami as a result of illegal mining (de Abreu et al., 2024), for example.

When discussing the Venezuelan population living with HIV there is no evidence to suggest that they are targeted specifically due to race or class, though sexuality may be a factor. In Latin America, HIV is largely concentrated amongst MSM (men who have sex with men) (García et al., 2014), a community marginalised and persecuted in Post-Chavez Venezuela (Kessler, 2023). As such, there may be some evidence to suspect a targeted politics of death, a condition suggested in Mbembe’s theorising. However, as policies that lead to ART resistance do not produce corpses (at least not for a time) this situation cannot be subsumed under necropower, but instead one must consider thanatopolitics, as earlier noted. Developing this nuance further, the scenario discussed in this paper is all played out at the level of the cell, where drugs and the virus interact with multiple different cell types though most notably white blood CD4 cells, which coordinate immune system response. Here, mortality refers to the slow death of CD4 cells, often long before the whole organism becomes deceased. As such, it is a form of governance and pharmaceutical politics that is played out in the micro and, as we are dealing with a virus, the microbiological.

Paxson (2008) coined the term ‘microbiopolitics’ to account for the social governance that human society had over microbial life, with particular focus on the changing ways that society lives with and alongside bacterial microbes. Borrowing the root notion of studying human governance over the life of microbes, I develop these ideas to offer the term ‘microthanatopolitics’, to refer to the ways in which human society can exert governance over the death of individuals through enacting health policies that explicitly unfold at the level of the microscopical cell. HIV is a virus so we cannot see it as microbial ‘life’, however the term microthanatopolitics need not be strictly restricted to Paxson’s original framework, nor specifically to Agamben’s thanato-politics as exclusionary to Mbembe’s necropolitics. I suggest that the term may apply to any situation whereby a politics of death is focused on cellular bodies, rather than full organisms to which the term originally pertains.

Politics that actively contribute towards the development of ART resistance do contribute towards death at a cellular level, yet this may not be immediately visible. As noted by HIV activist Jhonny above, this can indeed be seen as a form of genocide, but one that is initially cellular and drawn-out over time. As such, and for theoretical clarity, I maintain use of the root concept ‘thanatopolitics’ when creating the new term, whilst acknowledging Mbembe’s important theoretical development of ‘necropolitics’. As ‘microthanatopolitics’ is a new term, it should be stated that it incorporates conceptual viewpoints from both thanato-politics (death as concept) and necropolitics (corpse-production as a result of geo-political and colonial structures of hierarchy) and need not be tied to only the original ‘thanatopolitics’.

To develop this further, the paper will first review Venezuela’s past and present situation regarding ART provision and availability, showing how scarcity has increased as the crisis has developed, resulting in periods of ART disappearance and limited options. It is crucial to dedicate substantial space to this literature review, as the situation is complex and as-yet has not been covered in scholarship.

The relationship between ART availability and resistance will then be drawn to provide an understanding as to how the politics of pharmaceuticals can have very real consequences in the body, taking note of Lozano-Verduzco et al. (2021) suggestion that one must consider the intersectionality of migrants living with HIV and access to biomedicine.

Ethnographically this will be explored through discussion of lived experience of ART scarcity in Venezuela, and the resulting resistance that can, and has, developed. The discussion will then turn to the experience of Venezuelan migrants in Colombia, where working obligations and remittance priorities result in ART non-adherence and therefore potential resistance development, despite pharmaceutical availability in the host country. Through these lived examples, it will be possible to develop a discussion that addresses the politics of pharmaceuticals in Venezuela, microthanatopolitics, and how these are condemning Venezuelans living with HIV to potential ART resistance and therefore a future where limited treatment options are available. The paper takes a holistic approach to consider that microthanatopolitics not only be enacted by drug scarcity, but that a crisis that results in citizens’ foregoing medication to preference survival work in a neighbouring country could also be seen through this lens.

I will conclude that the Venezuelan ART supply may result in troubled futures of treatment option limitations, thereby compounding the challenges faced by this group long well into the future. Though Venezuela may have failed its citizens in this regard, it will also be considered how geopolitical relationships and especially the influence of the US has contributed towards this scenario, thereby highlighting how colonialities of power (Quijano, 2000) also impact politics of death in Latin America. Resistance testing and immediate provision of appropriate treatment regimens tailored to the individual will need to be a priority for Venezuelans living with HIV moving forward.

## 2 Background

### 2.1 ART in Venezuela

Antiretrovirals first became available and approved for treatment of HIV/AIDS by the U.S. Food and Drug Administration in 1987 (Warnke et al., 2007). By 1991, Venezuela began to provide free ART as part of the National HIV/AIDS-STI programme (*El Programa Nacional de VIH/-Sida-ITS*) that served the population free-of-charge through the national public health hospitals (Moloney, 2018). This was eight years prior to the presidency of populist leader Hugo Chávez, when Venezuela began to pursue socialist policies through the ideal of “21st century socialism” (though a socialist government would not be officially announced until 2007 (Cohen, 2019)). At the time of Venezuela’s initial ART rollout, socialist neighbour and future ally, Cuba, was still quarantining HIV positive individuals (Hansen and Groce, 2001). Neighbouring Colombia began providing ART in 1992 (Machado-Alba and Vidal, 2012), Brazil in 1996 (Parker, 2003), and Peru waited until 2004 to provide the medication (Soria et al., 2019). As such, when it comes to the provision of ART in Latin America, Venezuela was once a regional leader. However, since 2016, Venezuela stopped purchasing the necessary ART required to cover the population’s needs. This situation peaked between 2017 and 2018, when the government did not buy any ART at all (Acción Solidaria, 2019). Chávez’ successor, president Nicolás Maduro (2013-present), then rejected all foreign aid attempts, claiming that this was because “a country does not develop by accepting crumbs”; an assertion deeply tied to his denial that Venezuela in in crisis (France24, 2019). This scenario was exacerbated by the former acting president and US-backed opposition candidate, Juan Guaidó, publicly supported the acceptance of US and International humanitarian aid (Pozzebon, 2019). This power struggle contributed to further delays, until in late 2019 Maduro accepted medical aid, including ART from the Global fund (Acción Solidaria, 2019). However, the ART offered is limited, and it is estimated that only 10% of the population living with HIV are in receipt of ART as of 2023 (Johns Hopkins, 2023).

In existence there are more than 30 kinds of ART available that belong to 6 different classes, based on their mechanism of action (Pebody, 2021). Pharmaceuticals are given in combinations that are best suited to the patient, which can include considerations of whether or not a person is resistant to one drug class and/or type. Previously, 25 different types of ART were offered in Venezuela, though by the end of 2018 15 of these were reportedly out of stock (PAHO, 2018). With the intervention of the Global Fund, from 2019 onwards and as of 2024, the only combination now available in Venezuela is DLT (dolutegravir, lamivudine and tenofovir). This is a serious issue, as the DLT combination may not work well for everyone and may cause unwanted side effects or be ineffective. Considering the years when ART was not available at all and people went without, there may also be resistance to one or more components in DLT, meaning that patients are not being treated and the virus is not being suppressed. Some people who were previously prescribed any number of the other 25 drugs that used to be offered may have known resistance to DLT. The development of resistance to ART will be discussed further below. To note, that as Venezuela releases no health data, it is impossible to cite information about actual resistance in the country as of 2024.

Further, it is worth noting that although officially ART was free-of-charge prior to their 2016 disappearance and is presently free-of-charge with the Global Fund contributions, not everyone could or can access them due to the other hidden costs involved. For example, along with the health crisis, in 2012 came a 70% shortage of medicine and supplies meaning that necessary equipment like syringes and test kits were unavailable; not to mention the 15,000 doctors that fled due to the lack of resources and ability to care for their patients, resulting in limited personnel to attend to people (Wilson, 2015). Under these circumstances patients were asked to cover their own resource costs. This could be problematic for all those without a steady income who could not pay for the routine tests and bloodwork needed to receive the free ART, as the opening story of Germán and Iván showed. Because of this it is necessary to underscore that even if and when ART is technically available in Venezuela ‘free of charge’, it is not actually provided without any associated expenditure at all, and patients must pay for something for other. Alongside the general limited supply of ART, this offers further explanation as to why only 10% of those living with HIV are currently being treated.

Alongside data about the present scarcity, it is necessary to note that for many years, Venezuela provided a revolutionary healthcare system that catered to the whole population and not just those in the large cities or with the ability to fund private health insurance, as before (Brouwer, 2011). For example, a flagship of socialist Venezuela’s healthcare was the programme *‘Misión Barrio Adentro’*, which saw Cuban doctors enter into Venezuelan shanty towns (*barrios*) to provide multiple health services that the population had been previously denied, such as eye care and dentistry. *Barrio Adentro* was hailed as a realistic alternative to neoliberal healthcare where only the rich could achieve wellbeing (Muntaner et al., 2020), and Cooper (2019) claims that this and other socialist state healthcare programmes helped everyday Venezuelans to feel as though they were finally a part of the nation. As such, when discussing the corporeal politics of contemporary Venezuela, it is necessary to consider the geopolitical and post-colonial factors that have contributed towards the country’s current crisis, though an in-depth exploration of the same is outside the scope of this paper.

### 2.2 Developing ART resistance

Resistance to ART is considered as an emerging threat to controlling the global HIV pandemic (Beyrer and Pozniak, 2017), and resistance can either be transmitted or acquired. The WHO reported as many as 10% of newly diagnosed adults to already have resistance to the to the non-nucleoside reverse transcriptase inhibitors (NNRTI) drug class of ART (WHO, 2002). This is referred to as pretreatment drug resistance (PDR), and it happens because a person has been infected by a strain of HIV that is already resistant to one or more drug classes; ‘transmitted resistance’. Mother-to-infant transmission of ART resistant viral strains is also possible.

The three kinds of NNRTI drugs that patients most commonly have PDR to are nevirapine, delavirdine, and efavirenz (Joly and Yeni, 2000); none of which are found in the DLT combination currently offered in Venezuela. A global literature review found that PDR was more likely to be present amongst men who have sex with men (MSM), and sex workers (MacDonald et al., 2020). This is because these key populations share ART-resistant viral strains that circulate less in other key populations such as intravenous drug users. Data from Latin America shows that though Trans women are more likely to be diagnosed with HIV than any other group, the potential for PDR is the same as MSM (Trebelcock et al., 2019). However, the researchers also suggest likely under-reporting, being that genotypic resistance testing is not widely available in the region.

Genotype blood tests work by examining the genetic structure of a person’s HIV and uses this information to detect specific genetic mutations that are known to cause resistance to certain drugs and drug classes (Stanford Medicine, 2024). Such tests are expensive, and as such, are not undertaken with frequency. For example, Martínez-Cajas et al. (2013) report that in Colombia, medical guidelines do not recommend the use of genotype tests for treatment-naive patients or for people who experience a first therapeutic failure (when ART does not successfully increase CD4 count). Data from 2009 Venezuela report PDR at 11%, which is comparably higher than neighbours Brazil (1.4–6.5%) and Argentina (4.2%) (Rangel et al., 2009). In addition, over 50% with PDR displayed highly resistant viral strains at short intervals after initial diagnosis. This suggests that, even prior to the ART disappearance, Venezuelans were displaying resistance at higher levels, and with more virulent strains of HIV, than their neighbours. Though ART was still available in 2009, the compounding factors previously mentioned (such as inability to cover associated costs), may have contributed to this situation.

PDR is not the only scenario whereby ART will fail, and resistance can also be acquired. Acquired resistance occurs when the HIV virus is allowed to replicate and mutate while a person is taking (or is inconsistent with taking) ART. Human misuse of pharmaceuticals is a wide-spread issue in the development of antimicrobial resistance (WHO, 2023), and ART is no exception. Acquired resistance becomes a risk when people living with HIV (PLHIV) do not regularly take their ART, or if consistent ART is unavailable to them, though in more rare cases it can also occur when a person is taking their prescribed treatment. Acquired resistance can threaten the success of HIV treatment plans and increase the circulation of ART resistant viral strains (Crowell et al., 2021) and is a serious issue in the global management of the HIV pandemic. To tackle this, the WHO suggest an international, collaborative approach to providing successfully HIV treatment and switching regimens rapidly if treatment failure is detected (2022). Obviously, Venezuela is not doing this, and that can have global ramifications. In other contexts, ART has even been withheld over concerns that the population would not take the regimen ‘properly’ and could therefore develop resistant strains that they would then pass on to others. For example, in their ethnography on ART in Mozambique, Kalofonos (2021) describes how ART was restricted to some community members by NGO workers, on the belief that they would be irresponsible in taking the drugs and therefore develop resistance, which they would then pass along to others. Whilst this is arguably a paternalistic and troubling view that is entangled with structural factors such as racism and poverty, the cautious African rollout of ART underscores how large of a concern is ART resistance on a global scale. After all, HIV is borderless (Lindquist, 2005).

Yet, there are some rays of hope in this story too. Though resistance is more common to the NNRTI class of drugs, it has been shown that Dolutegravir has very high levels of viral load suppression. When people fail to follow their treatment regimen properly, there are fewer cases of acquired resistance to dolutegravir compared to other drugs (WHO, 2002), meaning it has a higher level of treatment failure ‘forgiveness’ than some other ART (Redzheb, 2023). Dolutegravir is part of the DLT combination on offer in Venezuela (Acción Solidaria, 2019), which may be promising in the face of involuntary treatment pauses and non-adherence. That said, one of the other DLT components, lamivudine, has already been shown to be ineffective against a prominent HIV mutant strain (Stanford Medicine, 2024). This goes to say that DLT is not fool proof against resistant strains of HIV, and should a Venezuelan PLHIV develop resistance, there would be no other treatment options available to them. Their ART would simply cease working as it should, putting them at risk for opportunistic infections and late-stage 3 HIV (AIDS). This is in addition to the fact that, without viral suppression, they could not reach undetectable status, and therefore run the risk of transmitting their resistant strain of HIV to others. Of note, once a person has developed resistance to a drug, this resistance applies to all drugs in that class. As mentioned earlier, there are only 6 classes of drugs (Pebody, 2021), and so it is important to avoid eliminating entire classes from one’s treatment options. If not, future treatment regimens may no longer be an option if needed. This highlights how a lack of ART availability may only last for a few short years of a person’s life but can have long-term consequences that may eventually render them unable to continue HIV treatment at all.

## 3 Methods

The paper is based on qualitative research collected during long-term ethnographic fieldwork from 2021 to 2024 with Venezuelans living with HIV in Colombia, in Bogotá and/or Santa Marta. The main research question addressed in this paper is the extent to which local and international politics have influenced the development of resistance to HIV medication in Venezuelans living with HIV, and the ways in which Venezuelans are negotiating ART scarcity and resistance to pharmaceuticals, or not.

During this time 40 testimonies of migrants living with HIV and/or utilizing the services of the HIV related NGOs in Bogotá and/or Santa Marta have been recorded, though it must be noted that not all of these testimonies spoke directly or indirectly about resistance. The majority of the participants came from the Venezuelan capital of Caracas (n = 21), followed by Zulia state (*n* = *5*), Carabobo (*n* = *3*), Trujillo (*n* = *2*), Anzoátegui (*n* = *2*), Merida (*n* = *1*), Sucre *(n* = *1*), Guyana (*n* =*1*), Aragua (*n* = *1*), Bolivar (*n* = *1*), Nueva Esparta (*n* = *1*), Lara (n = 1).

29 were cis-gendered men, 27 of whom identified as MSM, 6 were trans women, and 5 were cis-gendered women. The time spent living in Colombia ranged from 3 months to 8 years. Participants were aged between 21 and 47 years of age. 7 participants had health insurance in Colombia, whereas the other 33 were without insurance and would (presumably) need to access ART through an NGO. Participants were recruited through the International HIV NGO Aid for Aids, and the Colombian HIV NGO Fundación Eudes, who made initial introductions where the participant and I could discuss the project aims and use of the data. Participants were assured that their decision to participate had no bearing on ay relationship they had with the NGO(s). Snowballing recruitment was then utilized following initial introductions. All interviews were voice recorded except 2, where the interviewee declined recording. Interviews were transcribed and translated by the interviewer. Participants were gifted food and/or toiletry items as a thank you for their time. I travelled to a place and time of convenience to the participant, chosen by the participant. Data was not collected specifically about ethnicity; in the Venezuelan ‘café con leche’ ethnic context (Wright, 1993) where over 60% of individuals identify as Afro-descent, race can be perceived as fluid and dependent on context and circumstance. Initial testimonies were all collected in person, in Spanish, and all translations are mine.

In addition to the testimonies and of greater interest for this paper, interviews with HIV activists from and still living in Venezuela (*n* = 6), were undertaken from February–March 2024. The activists are from the states Caracas (*n* = *1*), Portuguesa (*n* = *1*), Zulia (*n* = *1*), Sucre (*n* = *1*), Miranda (*n* = *1*), and Nueva Esparta (*n* = *1*), and are all members of the Venezuelan network of positive people (*Red Venezolana de Gente Positiva* (RVG+)).

Interviews were undertaken virtually over zoom and transcribed and hand-coded for recurring themes. Only the interviews with HIV activists specifically focussed on resistance; in these cases, the transcripts were coded for sub-themes of resistance (ART shortage, Humanitarian crisis, HIV/AIDS in Venezuela, migration). Ethnographic fieldnotes were hand-coded for recurring themes. Ethical approval was provided by University REC committee. The study was limited by the fact that it is very difficult to obtain official resistance tests in both Venezuela and for the diaspora in Colombia. As such, some people who were resistant may not have known, and some with suspected resistance may not have been resistant. Further, few people interviewed knew the exact drugs they were on in the past, and now. Where the combinations are mentioned, it is either because the person knew, or because they showed me and this information was recorded. Without this exact information the following discussion cannot conclude on rates of resistance or drugs that are overwhelmingly involved, but only tell the stories of individuals whose experiences suggest a possible relationship to resistance.

Finally, my position is as a White woman living in Latin America but with an affiliation to a British University. As such, I acknowledge the necessity for dialogue and consultation with scholars and stakeholders from the communities of interest, in line with Savolainen et al.’s (2023: 1331) argument that it is not the biography of the researcher that is important, but collaboration and “the integrity of the collective process of truth-seeking”.

## 4 Results

### 4.1 Living ART scarcity in Venezuela

Growing up as an only child and spoiled with her parent’s attention, Mariette had always felt as though she was loved and cared for, though she noted feeling that something wasn’t right about her body from a young age. Her mother died when she was only five, back when she was still called Marcelo, and so she had no one to teach her about ‘feminine things’, as she called them. However, with her father’s full devotion and support, Mariette began living as a woman in her teenage years. “It’s quite unusual for a Caraqueño father to accept his, but my papá loves me so much”, she recounted to me during a conversation in 2022 about social acceptance for trans women in Latin America. He was also accepting when, at 17 years old, she was diagnosed with HIV in 2007. As she tells it, she had been enjoying her sexual freedom with different men she met on nights out at the gay nightclubs that were still operating in Caracas at the time, and wasn’t sure who had transmitted the virus to her. Mariette had made friends with other trans women, many of whom were sex workers, and they often spoke about the importance of HIV and STI tests for people in their community. Luckily for Mariette the HIV tests were still available in 2007, and she immediately began treatment through the public hospital in Caracas after her diagnosis.

However, the supply didn’t last, and for four years from 2013 to 2017 Mariette said she could not get a hold of any ART. Her hair fell out in chunks; hair that she had invested so much love and care into growing waist-length, an important part of her feminine identity. Her skin and eyes took on a yellow tinge, which she pointed out to me with a distinct air of sadness as we poured through Facebook photos from her ‘old life’ in Caracas. Mariette did not take any ART for the remainder of her time in Venezuela, and eventually made the decision to go to Bogotá in late 2017 in order to pursue a better future. The choice was not an easy one for her, as she did not want to leave behind her father; “I am *la hija única* (the only daughter), he needs me to take care of him now he’s getting older”, she told me. Ultimately, this reality was what made her decide to migrate to Colombia, in search of the promise of greater earnings and the chance to send some money home to her papá. Acquiring ART was also a factor, but at the time Mariette had no idea how this would be possible for a person who had migrated without any home or job to go to, as was her case.

It was within a new community of trans sex workers in Bogotá that Mariette learned about NGOs offering ART free of charge to Venezuelans. By this point she was going on four and a half years with no drugs, and she had to cover her wispy, balding scalp with a wig so as to continue feeling attractive – something very important to her. She knew what her combination had been in Venezuela – Atripla (efavirenz, emtricitabine, and tenofovir) – and so asked for this cocktail again when she had her first meeting at International NGO Aid for Aids’ clinic. But efavirenz no longer worked for Mariette, and after tests showed no increase in her CD4 count over six months, it was determined that she may have developed resistance. She was put on a combination of DET (dolutegravir, emtricitabine and tenofovir), which contains dolute-gravir, and so she would be less likely to develop resistance even if she did not take the regimen as required. Mariette knew information about dolutegravir and was the one who first alerted me to its hallowed status amongst HIV-positive Venezuelan migrants as ‘the best’ and supposedly almost resistance-resistant. In Mariette’s case, the ART resistance incited by the lack of options in Venezuela has found a solution for now, not least because she is currently residing in Colombia where there are more options. Had she stayed in Venezuela, she would never have had that recourse.

For those that have not migrated, the rising concerns over ability to get a hold of any ART at all in Venezuela has also affected people’s behaviours in taking their medication, even unwittingly contributing to their own potential resistance over fear of running out. As forty-nine year old activist Danilo from Miranda state noted, he meets many individuals who say they only take the DLT every other day so that they can stretch out the pills. However, this kind of spacing is not recommended and can contribute towards the development of resistance as well – but people are now too afraid that they won’t be given more ART that they are rationing what they have. Scarcity in Venezuela is not limited to ART, but also routine and specialised tests, as fifty-seven year old activist from Portugesa State, Yamaira, told me: “that one [DLT] can’t be taken by pregnant women, we can’t get the routine tests either [for viral load and CD4 counts], so we can’t check even if it’s working”. Yamaira runs a small NGO that helps support young families, and was particularly concerned that ART should be paused during pregnancy as this could increase the chances of perinatal transmission. Indeed, this is another issue of concern, and as she mentions, the lack of bloodwork tests available mean that people would be unaware if their medication is actually having the desired effect or not, or if they have become resistant. This reality condemns Venezuelans with HIV to continue with a treatment option that may not serve them, a reality that was not always the case. Yamaira said that when Chavez was still alive, things were “beautiful” – she had voted for him in the first election, and hadn’t experienced any ART availability issues up until the crisis hit under Maduro, But now, she says, “the government forgot us”.

### 4.2 Migrant struggles and non-adherence to ART

Whilst a government literally withholding precious medication may result in resistance, there are also other processes and experiences that lead people to neglect ART even when available, which also may lead to eventual resistance. Though this may not be the overtly direct work of a state, decades of policies that have led to widespread poverty and forced out-migration alongside remittance necessities do indeed contribute towards behaviours that encourage ART resistance in the long run.

One such example is that of Diego, a twenty-two-year-old MSM who theoretically has ART available to him but does not take it consistently. Having lived in Bogotá for the last three years, Diego is well aware of the free ART on offer at numerous NGOs and had previously accepted the pharmaceuticals from both Aid for Aids and Aids Healthcare International depending on who was offering more convenient appointment slots at the time. He said he’d managed to maintain the same combination through both NGOs, though Diego never told me which drugs he was taking in Colombia. He arrived in Bogotá already diagnosed with HIV, and said he had been taking DLT more or less consistently until his departure from Venezuela. Diego had been living with his family in the Caribbean city of Maracaibo, where he and his two brothers undertook odd jobs here and there (“*cualquier cosa*”), such as construction work around the neighbourhood, to support the family income. However, when Diego’s father died during the COVID-19 pandemic in 2020, he and the eldest brother decided to migrate to Colombia in order to earn the money now absent from the family, alongside their father. The remittances were sent to support their mother, two sisters, and younger brother who stayed behind, and Diego saw it as extremely important that he work hard to do this consistently. Luckily, he and his brother had excellent carpentry skills learned from their father and uncles, and found quick work making wooden benches to supply local restaurants in the Soacha area of the Colombian capital. This kind of work is commission-based, and the more benches that Diego produced, the more money he could make and send home. As he told me, this left very little time to schedule an NGO clinic visit; they operated mostly in the mornings when the boss was in the carpentry workshop, and it was hard to justify the 4-h round trip to pick up his ART. Moreover, ART is famous for producing gastrointestinal issues, and Diego felt that he could not lose time working for being incapacitated over acid reflux or diarrhoea. He couldn’t tell me how many times he’d missed his ART collections in the last three years, but he admitted that he was not taking the drugs with any regularity. I did ask if he was concerned about developing resistance, but he responded that this (and other HIV related worries) were simply not at the foreground of his mind, as he needed to send money home.

A similar story comes from Layla, a thirty-year old cis-gender woman from Valencia who works selling *tinto* coffee on the morning streets of Bosa in Bogotá. Layla also worried about sending remittances home to her elderly mother, but it wasn’t necessarily her desire to work all hours of the day that restricted her ART consumption. Instead, her role required that she work early mornings until lunchtime – prime time for Bogotanos to crave a caffeine fix. By the afternoon the *tinto* trade goes way down, and she wouldn’t make enough to survive if she gave up her morning routes. Yet, appointments at the NGOs she knew about were all in the morning. She could barely make them, and in the five years she’d been in Bogotá, she said she missed perhaps every third collection visit. Though there are other NGOs that may provide ART in Bogotá, in those with whom I worked and with whom interviewed migrants mentioned, there was limited appointment availability (though new online consultations were being trialled at the time of writing).

Others also skip medication even though they may have the pills, as they simply forget to take it. For example, at twenty-seven Renata already has five children, and she believes they are really a handful for her on her own. The youngest three-year-olds are twins, and Renata’s husband abandoned them all for another woman six months before I interviewed her in November 2023. She had the diagnosis for around a year, and in theory was able to make it to the appointments so long as the children either went to school or came along without a fuss. But she was so tired, she told me, that she kept forgetting to take the ART. Sometimes she’d ‘double up’ the next day, like she would do with the contraceptive pill if she forgot that – though she joked that this method clearly didn’t work either or she wouldn’t have so many children. In her case, the other demands of life kept getting in the way of her remembering to take the drugs, and in the way of her prioritising herself and her own health.

Although migrants, such as those cited above, may think that it is acceptable to take the drugs now and then, this in itself is an issue. As the Director for Aid for Aids Latin America told me, it would be better for them to take nothing at all, rather than sporadically take drugs, especially if they are combining different combinations. In taking nothing, at least they would not develop resistance, and could be put on a suitable regimen when they felt able to follow the treatment as prescribed. In taking different medications haphazardly, they may instead unwittingly be building resistance to the ART that is available to them in Colombia.

## 5 Discussion

A lack of ART availability and the general dearth of healthcare available in Venezuela means that the HIV community face tough decisions and the potential development of resistance. Yet, as noted, death is also not an immediate result of ART scarcity. A person can live with untreated HIV for up to 10 years before it progresses to late-stage HIV/AIDS, at which point there may be another 3–4-year window of treatment opportunity before death (UNAIDS, 2024). As such, any restriction of ART does not therefore equate with immediate mortality for Venezuela’s HIV positive population – in theory, if Venezuela simply provided no ART at all then some people may still live for upwards of a decade before needing intervention. Of course, this is an over-simplification of the reality, especially considering that economic poverty and the increase of opportunistic infections are related (Masanjala, 2007), meaning that a lack of ART may result in more rapid advancements to AIDS in a resource-impoverished Venezuela. Yet, speaking from a pharmaceutical politics perspective, it remains that it is more advisable to take nothing and wait so as to avoid the development of resistance, than to sporadically take different combinations of ART without any regularity. It is the latter that Venezuelans have been subjected to, meaning ART resistance becomes a real possibility later down the line.

People like Mariette and those in the communities that Danilo supports are developing resistance to certain ART drug classes due to current pharmaceutical politics in Venezuela. Whilst Mariette had the good fortune to arrive to a country where different drugs were available to her and this issue could be sidestepped, she has nevertheless lost a valuable defence in the existing possibilities to support healthy living with HIV, as would have many others. In turn, those with resistant strains of HIV are then at greater risk of infecting others with the virus (WHO, 2023). As noted, there are practically no condoms nor sexual health education programmes in Venezuela (Alabadejo, 2018), and so limited public health knowledge about prevention of HIV and other STIs. This is a perfect storm whereby those already with HIV may develop resistance to drugs, and those without HIV may be at risk of being infected with resistant viral-strains. Ultimately, this contributes towards the development of late-stage HIV/AIDS, and potentially, death.

As has been noted earlier, the politics of death (thanatopolitics; necropolitics) concern power over which groups of people are exposed to harm, with thanatopolitics more focussed on the concept of death, and necropolitics arguing for the production of literal corpses following discrimination based on colonial hierarchies. However, the scenario explored in this work is more nuanced than conceptual death or literal exterminations. I argue what is happening is at a cellular level; it takes a number of years for the HIV virus to multiply in the blood, to attack and kill precious CD4 cells, and to eventually bring down the entire organism. There is a politics of death the whole time, but it is the gradual death of CD4 cells rather than the immediate death of a person. It is this reality that necessitates the use of a new term, microthanatopolitics, to understand conceptually a politics of death where no death or dead bodies are immediately observed.

The microthanatopolitics of ART in Venezuela extend beyond ART scarcity. For example, one can consider the economic and financial crisis that has forced millions of Venezuelans to leave in order to survive as another form of this micro ‘politics of death’, as this too contributes towards situations whereby HIV positive people experience ART scarcity and associated immune cell death even where it is theoretically available. As the stories of Diego, Layla, and Renata demonstrate, the Venezuelan crisis does not only affect those living with chronic conditions. Family responsibility and caring duties follow migrants regardless of their HIV status. For example, remittances form a significant portion of Venezuelan’s survival, reaching US$4.2 billion in 2022, with an estimated 29% of Venezuelan households in receipt of financial support from family abroad (The Dialogue, 2023). These financial contributions positively impact food security (Stampini et al., 2021), healthcare access, and pension support for the elderly (Cuadros-Meñaca, 2020).

In addition to working to send remittance payments home, Venezuelans are also filling ‘undesirable’ job roles in host countries and boosting local economies (Taylor, 2024); the migrant workforce is very large, productive, and apparently willing to undertake employment in positions that locals do not want (such as selling coffee on the street, or low-pay manual labour, for example). Undesirable roles and remittance payments have become a necessity for survival, in no small part due to the dearth of work and money back home where people cannot even fulfil their basic needs (Caraballo-Arias et al., 2018).

As such, even when ART is available to migrants in Colombia, it can be seen that this is not a simple case of choosing to accept the drugs or not. Like all Venezuelan migrants, people living with HIV need to earn money for themselves and to send home, and this may impact their ability to regularly adhere to their treatment regimen. Importantly, treatment failures can have future ramifications.

At its worst, ART resistance reduces the options available to people living with HIV, meaning fewer treatment choices and the possibility that, in the most extreme cases, no drugs would suppress the virus. However, there are also more immediate concerns such as a potential inability to avail of the new injectable ART, for example. In Latin America this treatment option is currently only available in Argentina (Pebody and Hayes, 2024), however should eventually be a global option. The injection is given every six months and is comprised of cabotegravir (same class as dolutegravir) and rilpivirine (NNRTI class). As noted earlier, NNRTIs are the class of drugs that people are most likely to have resistance against (WHO, 2023). If a person has resistance to one of the components, then they cannot take the injection. As previously stated, the NNRTI class includes the drug efavirenz, and so for example, Mariette would not able to take the injection even if it were available in Colombia and/or Venezuela. That fact is a direct outcome of the ART scarcity whilst she was in Caracas.

The introduction of the ART injection would be an incredible support to marginalised migrant communities and Venezuelans living with HIV alike-migrants would only need to find the time to attend an appointment every six months, thereby being able to fulfil their work requirements whilst taking ART. It overcomes the issue of forgetting or avoiding taking the pills. Further, it would be of support for resource-poor Venezuelans who cannot afford the routine bloodwork every three months. However, the option is closed to those with NNRTI resistance; the inequality intersects so that a helpful ART option on the horizon may be incompatible with Venezuelans before it has even arrived. As such, it can be seen how developing resistance to ART drug classes is not simply an issue of switching pills, or migrating somewhere where you could do this, but may dictate future possibilities for those that already have limited choices of treatment.

It is also very important to state that whilst here the discussion has centred on treatment breaks as an issue for resistance, more immediately an ART treatment failure increases the risks of serious illness and death (Pebody, 2021). For example, the SMART study (Strategies for the Management of Antiretroviral Therapy) found that episodic ART use resulted in a significantly increased “risk of opportunistic disease or death from any cause” (2006: 2283). This result is clearly a more immediate example of a politics of death (and possibly necropolitics). However, in the absence of data from Venezuela, it is also difficult to comment on this scenario within the country.

Though this paper has centred on the localised gaps in Venezuela’s healthcare system and the State’s failure to provide necessary medical care, it is also important to place this within a wider geopolitical context, which this final section of the discussion will seek to do.

As was earlier noted, Venezuela was once a regional leader in ART provision, and developed community-minded healthcare that was accessible to wide swaths of the population during the presidency of Hugo Chavez. The crisis that ensued is complicated, and as Buxton (2021) suggests, there are fierce contestations over the roots of the issues. Notable factors include the transition from a democratic to an authoritarian government that involved mismanagement of funds (Bull and Rosales, 2020a), but also external factors such as falling oil prices (upon which Venezuela depended) and loss of political allies across Latin America due to the resurgence of the political right (Buxton, 2021). Critics point to the US sanctions and embargos that have impacted Venezuela’s economy and oil exports as a significant factor in political destabilisation (Bull and Rosales, 2020b). When considering not only the effect on individuals living with HIV but also the wider context in which a politics of death could emerge in the first place, it is therefore vital to place Venezuela, a post-colonial Latin American nation, within its geopolitical and historical perspective and contemplate how the Global North has, and continues to have, influence on outcomes at the personal, medical level.

Of importance, it is the Global Fund that is providing the (limited) ART that is currently available in Venezuela, and the US are the Global Fund’s largest donor (Global Fund, 2024). At the same time, US-backed embargos have influenced a situation whereby Venezuela has reduced economic power and therefore ability to purchase and supply ART of its choosing. This scenario speaks to a long-standing issue of paternalism in Western humanitarian aid (Eggen and Roland, 2013), but further, underscores the role that the Global North has played in the resulting situation described in this article. As such, it can be argued that the microthanatopolitics of Venezuelan ART scarcity and drug resistance is as much influenced by powerful institutions of the Global North as it is by the current Venezuelan government.

## 6 Conclusion

Amongst the wider social, economic, and political crisis that Venezuela is undergoing, those living with HIV are an important population of consideration, about whom scant attention and research has been developed thus far. If one looks superficially, then ART is now being provided in Venezuela and the problem may seem to be solved-why focus on people living with HIV when ART is available when, for example, infant deaths soar in the country (Garcia et al., 2024) or vaccine-preventable diseases have resurged (Bagcchi, 2021)? Through the careful discussion of how ART resistance develops, a review of Venezuela’s recent ART history, and reporting of lived experience, this paper has argued that we must look beyond the surface and question how local health policy and humanitarian aid may work together to (purposely, or not) contribute towards mass-ART resistance and in so doing, limit the treatment futures of Venezuelans living with HIV at home and abroad. Taking inspiration from both thanatopolitics and necropolitics, the framework of microthanatopolitics can help us to understand how a politics of death may affect people even where immediate death is not observable. Though this paper has focussed on Venezuelans living with HIV, the notion could be extended to any population living with HIV in post-colonial contexts whereby ART provision is only partial, and resistance may develop. Moving forward, testing for resistance and an immediate provision of appropriate treatment regimens, tailored to the individual, will need to be a priority for Venezuelans living with HIV both at home and abroad.

## Data Availability

No data was used for the research described in the article.
